# CD161 expression defines new human γδ T cell subsets

**DOI:** 10.1186/s12979-022-00269-w

**Published:** 2022-02-22

**Authors:** Amali Karunathilaka, Samuel Halstrom, Patricia Price, Michael Holt, Viviana P. Lutzky, Denise L. Doolan, Andreas Kupz, Scott C. Bell, Rachel M. Thomson, John J. Miles, Champa N. Ratnatunga

**Affiliations:** 1 Department of Microbiology, Faculty of Medicine, University of Peradeniya, Peradeniya, Sri Lanka; 2 School of Medicine, Curtin University, Western Australia, Australia; 3 The Prince Charles Hospital, Brisbane, Queensland Australia; 4 Gallipoli Medical Research Foundation, Brisbane, Queensland Australia; 5 QIMR Berghofer Medical Research Institute, Brisbane, Queensland Australia; 6 Australian Institute of Tropical Health and Medicine, James Cook University, Cairns, Queensland Australia; 7 Centre for Molecular Therapeutics, James Cook University, Cairns, Queensland Australia; 8 Faculty of Medicine, University of Queensland, Brisbane, Queensland Australia; 9 Division of Infection and Immunity, Cardiff University School of Medicine; Systems Immunity Research Institute, Cardiff University, Cardiff, UK

**Keywords:** Cellular immunity, CD161, γδ T cell, γδ T cell subsets, γδ T cell multifunctionality, High dimensional flow cytometry, Unsupervised clustering, FlowSOM, Immune checkpoint, Bronchiectasis

## Abstract

γδ T cells are a highly versatile immune lineage involved in host defense and homeostasis, but questions remain around their heterogeneity, precise function and role during health and disease. We used multi^−^parametric flow cytometry, dimensionality reduction, unsupervised clustering, and self-organizing maps (SOM) to identify novel γδ T cell naïve/memory subsets chiefly defined by CD161 expression levels, a surface membrane receptor that can be activating or suppressive. We used middle-to-old age individuals given immune blockade is commonly used in this population. Whilst most Vδ1^+^subset cells exhibited a terminal differentiation phenotype, Vδ1^−^ subset cells showed an early memory phenotype. Dimensionality reduction revealed eight γδ T cell clusters chiefly diverging through CD161 expression with CD4 and CD8 expression limited to specific subpopulations. Comparison of matched healthy elderly individuals to bronchiectasis patients revealed elevated Vδ1^+^ terminally differentiated effector memory cells in patients potentially linking this population with chronic proinflammatory disease.

**Supplementary Information:**

The online version contains supplementary material available at 10.1186/s12979-022-00269-w.

## Background

γδ T cells comprise only 1.5–3% of circulating T cells in humans [1] but 50–70% in ruminants and birds [2, 3], suggesting a pivotal role in host survival. Importantly, in humans, γδ T cells account for 60% of T cells in decidual tissues, 58% of T cells in the intestinal mucosa [4], 43% of T cells in lamina propria, and 15% of T cells in the skin. The protective nature of γδ T cell have been shown in pathogen defense of the skin, lung, intestines and circulation [5], defense from cancer [6], repair of the skin, epithelium, lung and gums as well as regulation of pregnancy, bone health, glucose levels, lymphoid stress and neurological memory [5, 7, 8]. These cells can be directly cytotoxic or recruit Th1, Th2, NK cells, B cells, macrophages, eosinophils and basophils [7].

Traditionally, γδ T cells existed as three populations based on δ chain expression. Vδ1 cells predominantly reside in mucosal sites and epithelia, while Vδ2 cells comprise 50–90% of γδ T cells in the circulation and are both effectors and antigen-presenting cells (APCs). Vδ3 cells are rare in circulation but enriched in the liver, notably in individuals with an infection or cancer [9]. As γδ T cells are less likely to cause graft-versus-host disease (GvHD) than their αβ counterparts, there has been intense interest in leveraging adoptive γδ T cell therapies for cancer [10] and infectious disease [11].

CD161 is a C-type lectin-like membrane receptor expressed at high levels on NK, NKT, Th17, Mucosa Associated Invariant T cells (MAITs), non-MAIT CD8^+^, tissue-resident memory cells (TRMs) [12–14], αβ CD4^+^ T cells and CD8^+^ T cells [14]. CD161 levels increase when αβ T cells enter tissues [14]. The ligand for CD161 is the lectin-like transcript 1 (LLT1). CD161/LLT1 engagement inhibits NK cell function but can inhibit or enhance αβ T cell function depending on the co-stimulatory molecules involved [14]. The inhibitory capacity of αβ T cells can be neutralized by blocking CD161 [15]. CD161 expression on γδ T cells is associated with enhanced IFN-γ and IL-17 [16, 17] production and enhanced endothelial transmigration [18].

Bronchiectasis is a chronic lung condition, defined as the abnormal, irreversible dilatation of the bronchi, where the elastic and muscular tissues are destroyed by acute or chronic inflammation and infection. This damage impairs the natural drainage of bronchial secretions, which can become chronically infected. Unless appropriately managed, the combination of repeated respiratory infection and chronic inflammation results in progressive lung damage [19]. While neutrophils and macrophages contribute to the pathogenesis of bronchiectasis, the involvement of γδ T cells has not been examined. However, γδ T cells are crucial for the killing of virus-infected cells and bacterial infection immunity, including Klebsiella and Pseudomonas infection, where γδ T cells mediate protection through IL-17A production [20]. It is likely γδ T cells play a significant role in bronchiectasis pathology due to chronic inflammation driven by repeated infection.

We performed a high-dimensional flow analysis with dimensionality reduction and unsupervised clustering of human γδ T cells from middle aged to elderly healthy individuals revealing a novel cell subset network, distorted in bronchiectasis.

## Methods

### Specimen collection and processing

Blood samples were obtained from 22 healthy adults (7 males, 15 females, aged 64.6 ± 8.8 yrs) and 11 bronchiectasis patients (8 males, 3 females; aged 74.8 ± 8.9 yrs). Patients were significantly older than the controls (Students *t*-test, *p* = 0.005), while the gender ratio was similar (Fishers exact test *p* = 0.99). Approval was obtained from the ethics review boards of Greenslopes Private Hospital (GSH), Prince Charles Hospitals (TPCH) and the QIMR Berghofer (QIMRB) Medical Research Institute (QIMRB P2058 and GSH 12/12 and 14/14). Informed written consent was obtained from all participants. Whole blood was collected with EDTA anticoagulant tubes (BD Life Sciences, NJ, USA). Peripheral blood mononuclear cells (PBMCs) were isolated by Ficoll^−^Paque PLUS (GE Healthcare, IL, USA) by density gradient centrifugation and resuspended in RPMI-1640 containing 10% foetal calf serum with 10% DMSO for cryopreservation in liquid nitrogen.

### Flow cytometry

Cells were stained with LIVE/DEAD reagent (Invitrogen, MA, USA), anti-CD3 APC-eFluor780 (ThermoFisher, MA, USA), anti-CD4-BUV395, anti-CD8-BV786, anti-CD27-BUV737, anti-CD45RA-APC-H7 and anti-HLADR-BV650 (BioLegend, CA, USA), anti-CD161-PE, and anti-ϒδ TCR-PE-CY7 (BD Life Sciences, NJ, USA) and anti-Vδ1-FITC (Miltenyi Biotec, Bergisch Gladbach, Germany) (see Supplemental Table 1). Data were acquired on an LSR-Fortessa II, and BD FACSDiva Software (BD Life Sciences, NJ, USA).

### Flow data analysis

Gating was performed using standard singlet and viability gates. Positives were gated based on fluorescence minus one (FMO) controls. Manual gating was performed using FlowJov10.8(LCC, OR, USA). γδ T cells were divided into two populations based on Vδ1 expression. Each population was subgated based on CD45RA and CD27 expression to differentiate naïve and memory cells and then gated based on CD161, CD4 and CD8expression. Cytobank 5.0 (https://www.cytobank.org/, Beckman Coulter, CA, USA) was used (following quality control and data scaling) to gate live/CD3^+^/γδ TCR^+^cells. On manually gated γδ TCR^+^cells, Uniform Manifold Approximation and Projection (UMAP) analysis was performed using Vδ1, CD45RA, CD27, HLA-DR, CD161, CD4, and CD8 as clustering channels [21]. UMAP is a dimensionality reduction method which allows the observer to visualize high dimensional data in a low dimensional graph, providing meaningful cell clusters based on marker expression, which has been shown to be both reliable and robust. Automated clustering algorithm FlowSOM has been shown to perform better than other unsupervised methods in precision, coherence and stability and was therefore chosen for this exploratory analysis [22, 23]. Subsequent FlowSOM analysis (automated analysis) on the resulting UMAP was performed on Vδ1, CD45RA, CD27, HLA-DR, CD161, CD4, and CD8 expression, generating eight meta-clusters. For the purpose of identifying rare populations, FlowSOM analysis was set to 100 nodes as recommended in the reference protocol [24]. In FlowSOM, all cells in all samples are grouped together. This is followed by using multidimensional input data (all input channels) to generate an artificial neural network of cell nodes. Each cell is clustered to the node that most closely represents its expression pattern. Closely connected nodes are similar while distant nodes are dissimilar, thus forming a topological map of information without bias. Node size depends on cell number. Similar clusters are grouped into meta-clusters. Detailed phenotypic exploration of cells identified by these two independent methods was performed and findings validated using conventional biaxial gating from original flow data using FlowJo v10.8 (LCC, OR, USA).

### Statistics

Students *t* test and Mann Whitney U test was performed using IBM SPSS v20 (IBM), and Prism 9.1.1 (GraphPad Prism, CA, USA). *p*-values are shown. *p < 0.05, **p < 0.01, ***p < 0.001, ****p < 0.0001.

## Results and discussion

### The surface receptor patterns that defineVδ1^+^ and Vδ1^−^ memory cells

**Fig. 1:**
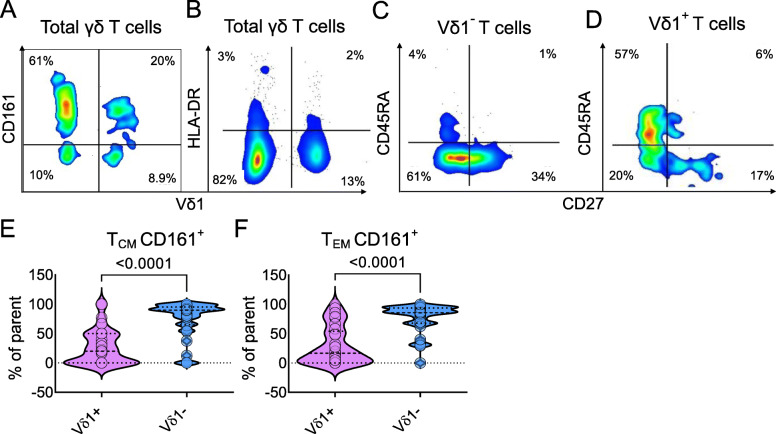
γδ T cells subsets exhibit distinct surface protein fingerprints. Total γδ T cells were gated on (**A**) CD161 and (**B**) HLA-DR versus Vδ1. Vδ1^−^ (**C**) and Vδ1^+^(**D**) T cells were gated on CD45RA versus CD27,. An example of biaxial density plots from one donor is shown. Comparison of CD161 expression on (**E**) T_CM_ cells and (**F**) T_EM_ cells in Vδ1^+^ and Vδ1^−^ populations showing elevated CD161 expression in Vδ1^−^ cells (*p* < 0.0001)

Flow-based analyses of PBMC samples from 22 healthy individuals revealed a mean γδ T cell (as a percentage of CD3^+^ cells), Vδ1^+^ and Vδ1^−^ (as a percentage of γδ T cells) of 8.6% (± SD 6%), 30.6% (± SD 27%) and 69.2% (± SD 27%) respectively which are similar to values published for a cohort that included elderly individuals [25]. Four distinct γδ T cell subsets were identified when gating on Vδ1 and CD161 (Fig. 1A), with enumerative means and ± SDs in Vδ1^−^CD161^+^cells (53% ± 30%), Vδ1^−^CD161^−^cells (12% ± 9%), Vδ1^+^CD161^−^(18% ± 23%), andVδ1^+^CD161^+^cells (16% ± 20%) determined. Four subsets were observed when combining Vδ1 and HLA-DR expression (Fig. 1B), with enumerative means and SDs in Vδ1^−^HLA-DR^−^cells (54% ± 28%), Vδ1^−^HLA-DR^+^cells (11% ± 13%), Vδ1^+^HLA-DR^−^cells (27% ± 26%), and Vδ1^+^HLA-DR^+^cells (8% ± 8) determined. Such high HLA-DR expression on γδ T subsets implies the operation of complex APC machinery for adaptive system function. CD45RA and CD27 were used to gate CD45RA^+^CD27^+^ naïve (T_N_), CD45RA^−^CD27^+^ central memory (T_CM_), CD45RA^−^CD27^−^ effector memory (T_EM_) and CD45RA^+^CD27^−^ terminally differentiated effector memory (T_EMRA_) in the Vδ1^+^ and Vδ1^−^populations (Fig. 1C&D). We observed few T_N_ in all subsets. Interestingly, the memory profiles of Vδ1^+^ and Vδ1^−^ populations were noticeably divergent. In Vδ1^+^population cells, there were mainly two population foci. One in the T_EMRA_ population (37% ± 28) and one in T_EM_ population (40% ± 22%) with some bleeding into T_CM_ population (18 ± 21%). In contrast, in the Vδ1^−^ population, there was a clearly defined population in T_EM_ cells (53 ± 20%) and a T_CM_ population (27 ± 18%) with some bleeding into the T_EMRA_ population (16 ± 21%). Comparison of CD161 expression between naïve and memory Vδ1^+^ and Vδ1^−^populations revealed a significant difference in both T_EM_ cells and T_CM_ cells (*p* < 0.0001), with Vδ1^−^ cells having the highest surface expression of CD161 (Fig. 1E&F).

### Dimensionality reduction and unsupervised clustering with self organizing maps (SOMs) reveal new Vδ T cell types

**Fig. 2:**
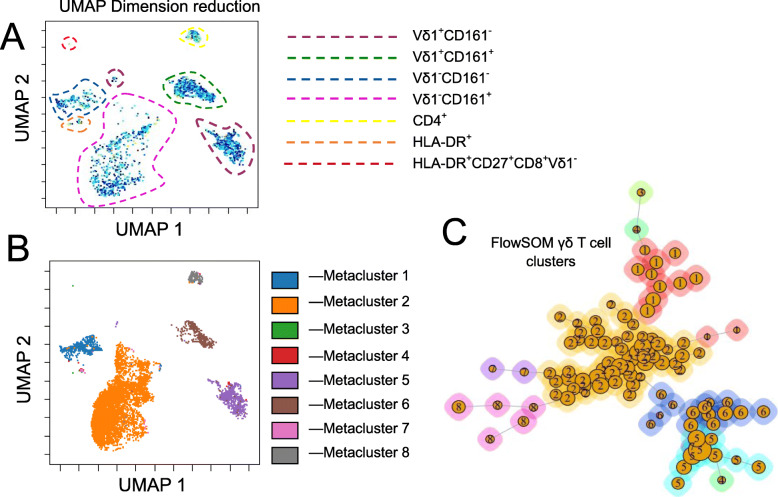
UMAP (uniform manifold approximation and projection) together with FlowSOM identifies phenotypically unique subsets of γδ T cells in healthy elderly individuals. **A** UMAP dimensionality reduction performed the whole γδ T cell population grouped cells into 8 islands when Vδ1, CD161, CD45RA, HLA-DR, CD27, CD8 and CD4 were used as clustering channels. **B** FlowSOM meta-cluster overlayed on UMAP plot indicating high degree of correlation between two independent automated analyses. **C** FlowSOM meta-cluster positions on the minimal spanning tree. The meta-cluster numbers given here represents numbers shown in panel (**B**) and Table 1

**Fig. 3:**
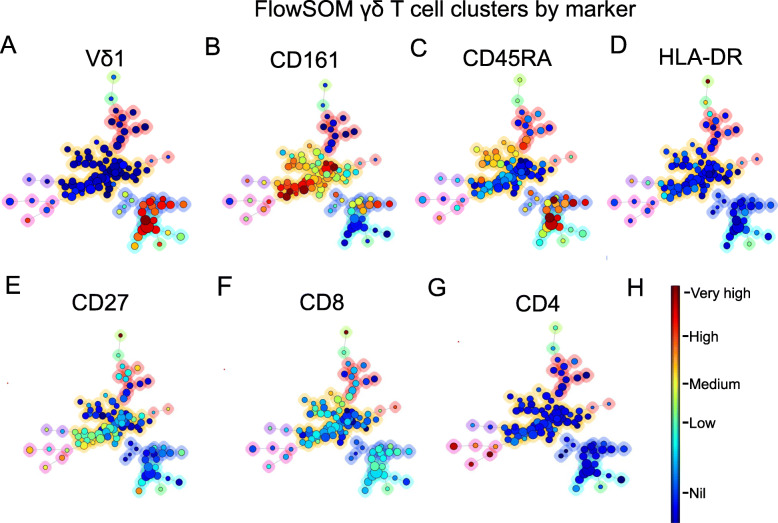
FlowSOM minimal spanning tree (MST) clustering identifies eight novel γδ T -cell subsets. Total γδ T cell flow data from middle aged to elderly healthy donors were clustered into 100 nodes with subsequent automated meta-clustering into eight γδ T cells subsets (meta-clusters) using Vδ1, CD161, CD45RA and, CD27, CD8 and CD4 clustering channels and the FlowSOM algorithm. The relationships between the nodes (which are most like each other) are shown by the spanning tree with similar nodes placed close together on the plot. Expression intensity of each clustering marker on the same spanning tree plot is shown in the six diagrams (**A**) Vδ1, (**B**) CD161, (**C**) CD45RA, (**D**) HLA-DR (**E**) CD27, (**F**) CD8 and **(G)** CD4. Dark red represents maximum expression, and dark blue represents no expression of the given marker as shown in the colour bar. A coloured halo around the node indicates the meta-cluster to which each node belongs. Expression levels of each marker in each cell meta-cluster is summarized in Table 1

We next performed the UMAP dimensionality reduction on manually gated γδ T cells, which clustered the population into eight islands (Fig. 2A and B). The four populations previously identified by manual gating based on CD161 and Vδ1 expression clustered to five islands, supporting that CD161 is a primary differentiating marker in γδ T cells (Fig. 2A). Three smaller islands were also seen; a CD4^+^ island containing both Vδ1^+^ and Vδ1^−^ cells, and two very small HLA-DR^+^ islands. Independent FlowSOM analysis on this data revealed similar results. Vδ1^+^ T cells primarily aligned with meta-clusters 5 and 6, showed a predominantly T_EMRA_ phenotype and were either CD161^+^ (meta-cluster 6) or CD161^−^ (meta-cluster 5) (Fig. 2C, 3A-G and Table 1.). Vδ1^−^cells, likely comprising Vδ2^+^ and Vδ3^+^ ϒδ T cells, aligned into meta-clusters 1–4, differentially expressed CD161with meta-cluster 2 being CD161^+^ while the others were CD161^−^ and expressed CD27 and CD45RA with subtype-specific patterning (Table 1). Meta-cluster 3, 4 and 7 consist of cells expressing HLA-DR, of which meta-cluster 4 contained Vδ1^−^ CD161^−^cells while meta-cluster 7 contained CD161^+^ Vδ1^+^ and Vδ1^−^cells. Meta-cluster 3 was the smallest meta-cluster and contained rare HLA-DR^+^CD8^+^CD27^+^ Vδ1^−^cells. Meta-cluster 8 contained Vδ1^+^ and Vδ1^−^cells that express CD4 while CD8 expression was limited to meta-cluster 3 and a small subset of meta-cluster 1 (Table 1). When FlowSOM meta-clusters were overlaid on the UMAP plot, the resulting clusters were almost identical (Fig. 2B), indicating a high degree of agreement between the two independent methods. Both methods show CD161 as a primary differentiating phenotypic marker for Vδ cell subsets. These data were validated using manual biaxial gating where the CD4^+^ γδ T cells (meta-cluster 8), HLA-DR^+^CD161^−^ (meta-cluster 4) and HLA-DR^+^CD161^+^ (meta-cluster 7) population means were 3.7% (SD- 2.4%), 3.3% (SD- 2%) and 6.4% (SD- 4.2%) respectively. The HLA-DR^+^CD8^+^CD27^+^ Vδ1^−^ cell population (meta-cluster 3) was relatively small (0.4%, SD- 0.4%) which corresponds the very small cell island identified as phenotypically distinct by UMAP analysis. This population, though small, expressing very high levels of HLA-DR, and CD27 may be a highly activated, functionally distinct subset. Overall, the pattern of marker expression on the eight clusters, as detailed in Figs. 2 and 3 and Table 1, show that CD161 plays a central role in the differentiation of γδ T cell subtypes. Moreover, CD4 and CD8 expressing γδ Tcells are relatively rare [26, 27].

**Table 1:**
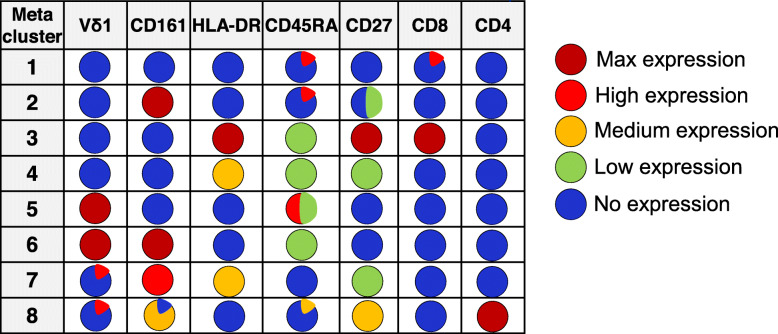
The cell surface landscape of ϒδ T cells subsets

### The γδ T cell subtype numbers are distorted in bronchiectasis

We then compared the γδ T cell subtype network in the circulation of healthy individuals with bronchiectasis patients. Since patients were found to be significantly older than the healthy controls (74.8 yrs. versus 64.8 yrs., *t-*test *p* = 0.005), to match the bronchiectasis patients age, five samples (age ranging from 51 to 59 years) from the healthy controls were removed for this analysis. Relative to controls, bronchiectasis patients showed higher numbers of Vδ1^+^ T_EMRA_ cells (*p* = 0.03). The two cohorts were similar in peripheral blood γδ T cell phenotype in all other aspects tested.

### Overview of γδ T cell subsets architecture

Recent high-dimensional and automated clustering studies have identified distinct γδ T cell subsets in circulation, differentially expressed in disease [28–30]. Here, we define eight γδ T cell subsets in healthy middle aged to elderly humans that depend primarily on CD161 expression. Conventional biaxial gating revealed Vδ1^+^ and Vδ1^−^T cell subsets in the blood contrast in phenotype. As Vδ1^−^ cells are likely to be the predominant Vδ2^+^ in circulation, these results show that the probable Vδ2^+^T cells are predominantly T_EM_/T_CM_ in elderly healthy individuals, consistent with previous reports [31] In addition, we showed that Vδ1^+^cells maintain a predominant population of T_EM_/T_EMRA_ γδ T cells. Peripheral blood T_EMRA_ Vδ2^+^ T cells are highly active in antibacterial and anti-tumor immunity [32]. With a predominant T_EMRA_ population, its possible Vδ1^+^ cells have a significant role to play in these scenarios as well. CD161 expression on T cells has been shown to characterize a unique functional phenotype across multiple cell lineages [33]. Moreover, CD161 expression on γδ T cells is associated with high expression of the transcription factor retinoic acid-related orphan receptor C (RORC) and IL23R, and IL17 production [17] The very high expression of CD161 on Vδ1^−^ T_CM_ and T_EM_ compared to their Vδ1^+^ counterparts indicate that these two cell subsets have divergent functional roles in peripheral blood. Recent data showing differential expression of immune checkpoint CD5 and activation marker CD28 on γδ T cell Vδ subsets supports this hypothesis [25]. The role of γδ T cells in bronchiectasis has not been characterized to date, although their role in lung defense against bacteria and other chronic disease has been described [20]. Whether the peripheral blood signature identified here is indicative of a more active γδ T cell response present in the lung is yet to be determined. Nonetheless, the increase in Vδ1^+^T_EMRA_ cells suggested an ongoing, vigorous response.

### Study limitations and future directions

To our knowledge, these data are the first to differentiate γδ T cells subsets in aged individuals. The γδ T cell compartment changes in abundance, Vδ composition and clonality with age [34]. Further work studying the phenotypical changes in γδ T cell subsets over the human life-course will help explain the divergent results observed to date. Looking beyond conventional subsets, we analyzed the γδ T cells for CD161expression using unsupervised clustering. Here FlowSOM revealed Vδ1^+^ cells were primarily T_EMRA_ and T_EM,_ where CD161 was differentially expressed. Vδ1^−^ subsets were primarily T_EM_ with a range of surface CD161 expression. Biaxial gating showed that the majority of both Vδ cell populations were HLA-DR negative while specific small subpopulations with high expression of this activation marker were present. This study looked at circulating γδ T cells, and tissue resident γδ T cells will likely present further subtypes.

A comparison of healthy individuals with bronchiectasis patients revealed a distortion in the γδ T cell subtype network with increased Vδ1^+^T_EMRA_. CD161 levels on γδ T cells and NKT cells do not change with age [35] (Supplemental Fig. 2). Little is known about HLA-DR expression in γδ T cells during aging, however, HLA-DR^+^αβ CD8^+^ Tregs are known to accumulate with age [36] and a reduction in HLA-DR levels would impede adaptive functions as HLA levels correlate with T cell priming and activation [37]. In γδ T cells, there is no change in CD27 expression with age [38]. An increase in T_EMRA_ cells, could result in a reduced capacity in pathogen control [39].

A limitation of the study is the age of the healthy controls (mean 64 yrs). This age cohort was selected due to relevance, with a peak incidence in cancer, lung infections and bronchiectasis. Validation of these results is required in other age groups to determine the temporal dynamics of γδ T cells. Future studies should also address the knowledge gap inCD161 and LLT1 signaling. In summary, we have defined eight novel γδ T cell subsets that show some distortion in bronchiectasis.

## Supplementary Information

**Additional file 1: Sup Fig. 1.** Gating strategy followed for biaxial gating. **Sup Fig. 2** CD161 expressing cell percentages in **(A)** total γδ T cell, **(B)** Vδ1^+^γδ T cell and **(C)** Vδ1^−^γδ T cell populations. CD161 expression shows no correlation with age in any of the analyzed subsets (R squared/ *p* value for each subset *R*^2^ = 0.133/*p* = 0.125, *R*^2^ = 0.027/ *p* = 0.465, *R*^2^ = 0.015/ *p* = 0.579 respectively). **Sup Fig. 3** Fluorescence minus one (FMO) staining controls for CD161, HLA-DR, CD45RA and CD27 are shown together with fully stained sample showing staining pattern on γδ T cells.

**Additional file 2: Supp. Table 1.** Flow cytometry staining panel.

## Data Availability

The dataset generated and analyzed during the current study are part of a more extensive unpublished study, and thus are not publicly available. However, manuscript data can be made available from the corresponding author (CNR) upon reasonable request.

## Abbreviations

APCs: Antigen-presenting cells
GVHD: Graft-versus-host disease
MAIT cells: Mucosa Associated Invariant T
TRM cells: Tissue resident memory
LLT1: Lectin-like transcript 1
QIMRB: Queensland Institute of Medical Research
BD: Becton Dickinson
PBMCs: Peripheral blood mononuclear cells
FMO: Fluorescence minus one
T_N_: Naïve T cells
T_CM_: Central memory T cells
T_EM_: Effector memory T cells
T_EMRA_: Terminally differentiated effector T cells
SOMs: Self-Organizing Maps
MSTs: Minimum Spanning Trees
UMAP: Uniform manifold approximation and projection
