# Supplementary figures and images for: CD161 expression defines new human γδ T cell subsets
Source: Immun Ageing. 2022 Feb 22;19:11. doi: 10.1186/s12979-022-00269-w (PMC8862246; doi:10.1186/s12979-022-00269-w)

Supp. Fig. 1

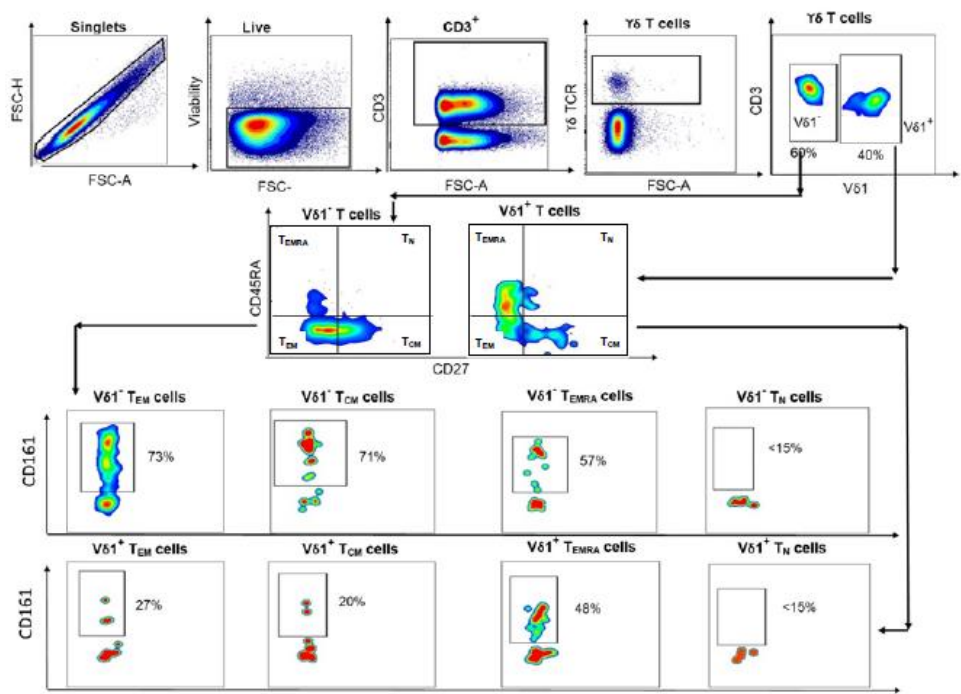

Supp. Fig. 2

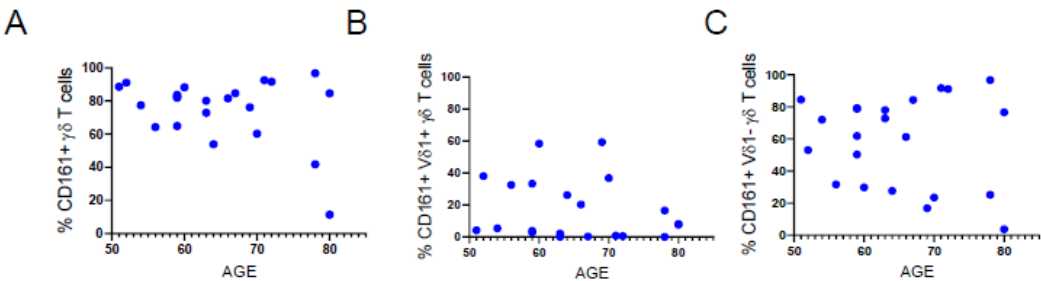

Supp. Fig. 3

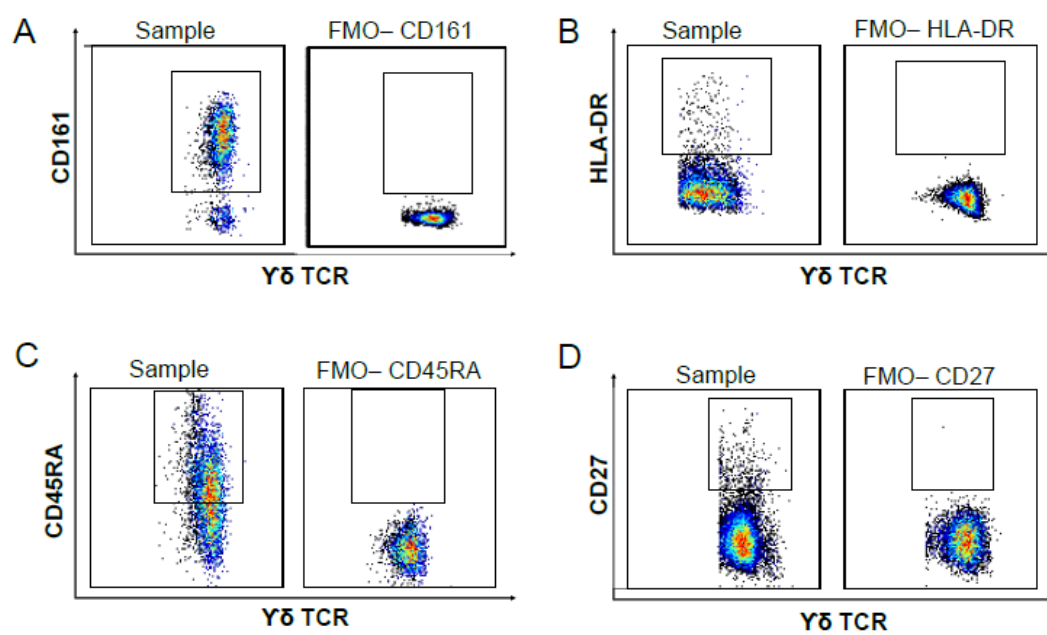

Supplement: Supplementary file 1 — Additional file 1: Sup Fig. 1. Gating strategy followed for biaxial gating. Sup Fig. 2 CD161 expressing cell percentages in (A) total γδ T cell, (B) Vδ1+γδ T cell and (C) Vδ1−γδ T cell populations. CD161 expression shows no correlation with age in any of the analyzed subsets (R squared/ p value for each subset R2 = 0.133/p = 0.125, R2 = 0.027/ p = 0.465, R2 = 0.015/ p = 0.579 respectively). Sup Fig. 3 Fluorescence minus one (FMO) staining controls for CD161, HLA-DR, CD45RA and CD27 are shown together with fully stained sample showing staining pattern on γδ T cells. [file 12979_2022_269_MOESM1_ESM.pdf]
