# Supplementary material for: CD161 expression defines new human γδ T cell subsets
Source: Immun Ageing. 2022 Feb 22;19:11. doi: 10.1186/s12979-022-00269-w (PMC8862246; doi:10.1186/s12979-022-00269-w)
Supplement: Supplementary file 2 — Additional file 2: Supp. Table 1. Flow cytometry staining panel. [file 12979_2022_269_MOESM2_ESM.docx]

Supp. Table 1. Flow cytometry staining panel

| Fluorochrome | Marker | Clone | Volume | Manufacturer |
| --- | --- | --- | --- | --- |
| BUV395 | CD4 | SK3 | 1. 5 | BioLegend |
| BUV737 | CD27 | L128 | 1. 2 | BioLegend |
| BV650 | HLA^-^DR | G46 | 1. 25 | BD Biosciences |
| BV786 | CD8 | RPA^-^T8 | 1 | BioLegend |
| FITC | Vδ1 | REA173 | 2 | Miltenyi Bio |
| PE | CD161 | 191B8 | 1 | BD Biosciences |
| PE^-^Cy7 | ϒδ TCR | 11F2 | 2. 5 | BD Biosciences |
| APCeflour 780 | CD3 | UCHT1 | 1. 5 | ThermoFisher |
| AF700 | Viability | ^-^ | 0. 1 | Invitrogen |
| APC^-^H7 | CD45RA | HI100 | 1. 2 | BioLegend |
